# plotsr: visualizing structural similarities and rearrangements between multiple genomes

**DOI:** 10.1093/bioinformatics/btac196

**Published:** 2022-04-15

**Authors:** Manish Goel, Korbinian Schneeberger

**Affiliations:** Faculty of Biology, LMU Munich, Planegg-Martinsried 82152, Germany; Department of Genetics, Faculty of Biology, LMU Munich, Germany; Faculty of Biology, LMU Munich, Planegg-Martinsried 82152, Germany; Department of Genetics, Faculty of Biology, LMU Munich, Germany

## Abstract

**Summary:**

Third-generation genome sequencing technologies have led to a sharp increase in the number of high-quality genome assemblies. This allows the comparison of multiple assembled genomes of individual species and demands new tools for visualizing their structural properties. Here, we present plotsr, an efficient tool to visualize structural similarities and rearrangements between genomes. It can be used to compare genomes on chromosome level or to zoom in on any selected region. In addition, plotsr can augment the visualization with regional identifiers (e.g. genes or genomic markers) or histogram tracks for continuous features (e.g. GC content or polymorphism density).

**Availability and implementation:**

plotsr is implemented as a python package and uses the standard matplotlib library for plotting. It is freely available under the MIT license at GitHub (https://github.com/schneebergerlab/plotsr) and bioconda (https://anaconda.org/bioconda/plotsr).

**Supplementary information:**

Supplementary data are available at *Bioinformatics* online.

## 1 Introduction

Third-generation sequencing technologies together with efficient phasing and scaffolding methods like Hi-C, trio-binning or gamete-binning have led to a sharp increase in the number of available haplotype-resolved, chromosome-level assemblies (Campoy *et al.*, 2020; Koren *et al.*, 2018; Zhang *et al.*, 2019). Chromosome-level assemblies allow the identification of small genomic differences (like SNPs and indels) as well as large structural rearrangements (SRs) like inversions and translocations. Therefore, they are considered the gold standard for genomic differences identification (Simpson and Pop, 2015).

Typically, the overall structure of individual genomes of a specific species is highly conserved because the genomes recombine (exchange chromosome arms) during sexual reproduction and thereby keep the same karyotype across different haplotypes. This introduces large syntenic regions between the genomes where recombination can occur without affecting the overall structure of the genomes. During genome comparisons, these syntenic regions (the ‘syntenic backbone’) can be identified. All remaining regions in the genomes are structural rearrangements by definition and can then be classified into inversions, duplications or translocations (here, we consider both intra- and inter-chromosomal relocations as translocations) based on their orientation and location in the genomes. Recently, we used these principles to develop SyRI, a tool to identify genomic differences in whole-genome assemblies of the same species (Goel *et al.*, 2019).

For better analysis of these genomic differences between multiple genomes, there is a need for accurate and intuitive visualization tools. Currently available tools can visualize large structural rearrangements between a pair of genomes (e.g. MUMmer, Ribbon, rearrvisr) or are designed for visualizing local variations like SNPs and indels in pan-genomes (e.g. tubemaps, ODGI) (Beyer *et al.*, 2019; Guarracino *et al.*, 2021; Kurtz *et al.*, 2004; Lindtke and Yeaman, 2020; Nattestad *et al.*, 2021). For the visualization of large structural rearrangements in multiple genomes, we have developed **plotsr** (**plot s**tructural **r**earrangements). plotsr uses the synteny between genomes to identify homologous chromosomes as well as to match the orthologous regions between the genomes allowing for efficient zooming in on specific regions. It is a simple-to-use yet flexible and powerful visualization tool. It can be used to compare multiple haploid genomes as well as different haplotypes of individual polyploid genomes. In addition, plotsr can mark specific loci as well as plot histogram tracks to show distributions of genomic features along the chromosomes.

## 2 Implementation

**Fig. 1.**
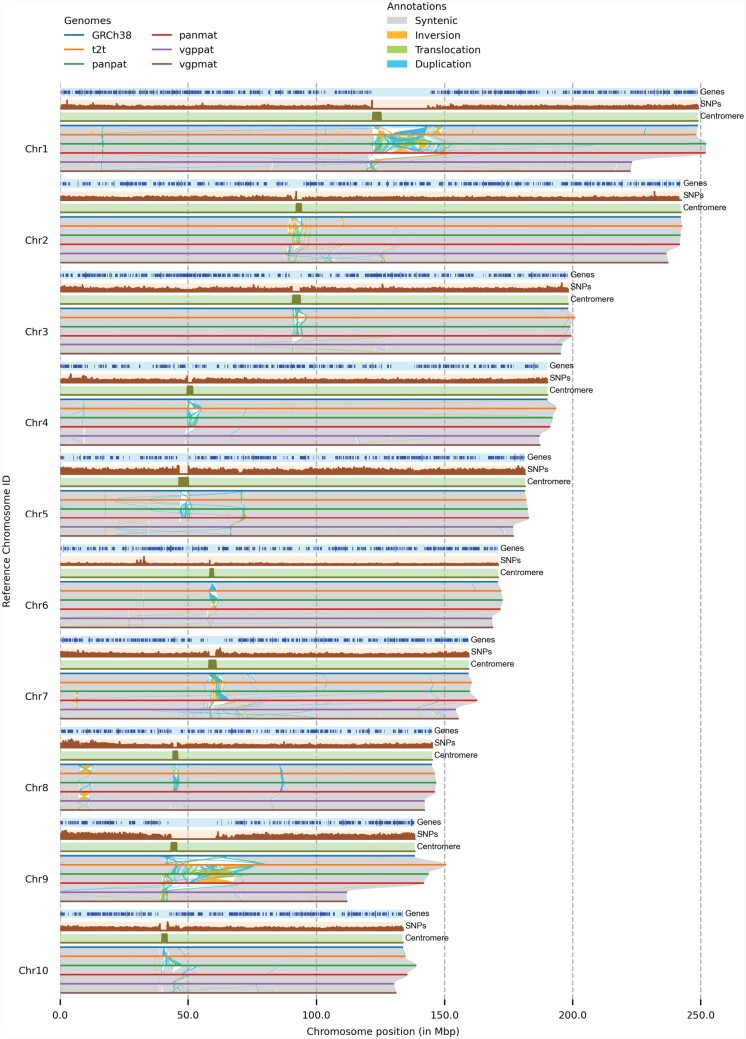
Visualizing structural rearrangements using plotsr. We used plotsr to visualize syntenic regions and structural rearrangements between 10 chromosomes from 6 human genomes. The visualization was created using plotsr without further modifications. Tracks for three genomic features: genes, number of SNPs and centromeric regions were included using optional parameters. In the genes track, smaller lines correspond to transcribed regions and longer lines represent coding-sequences (CDS)

**Fig. 2.**
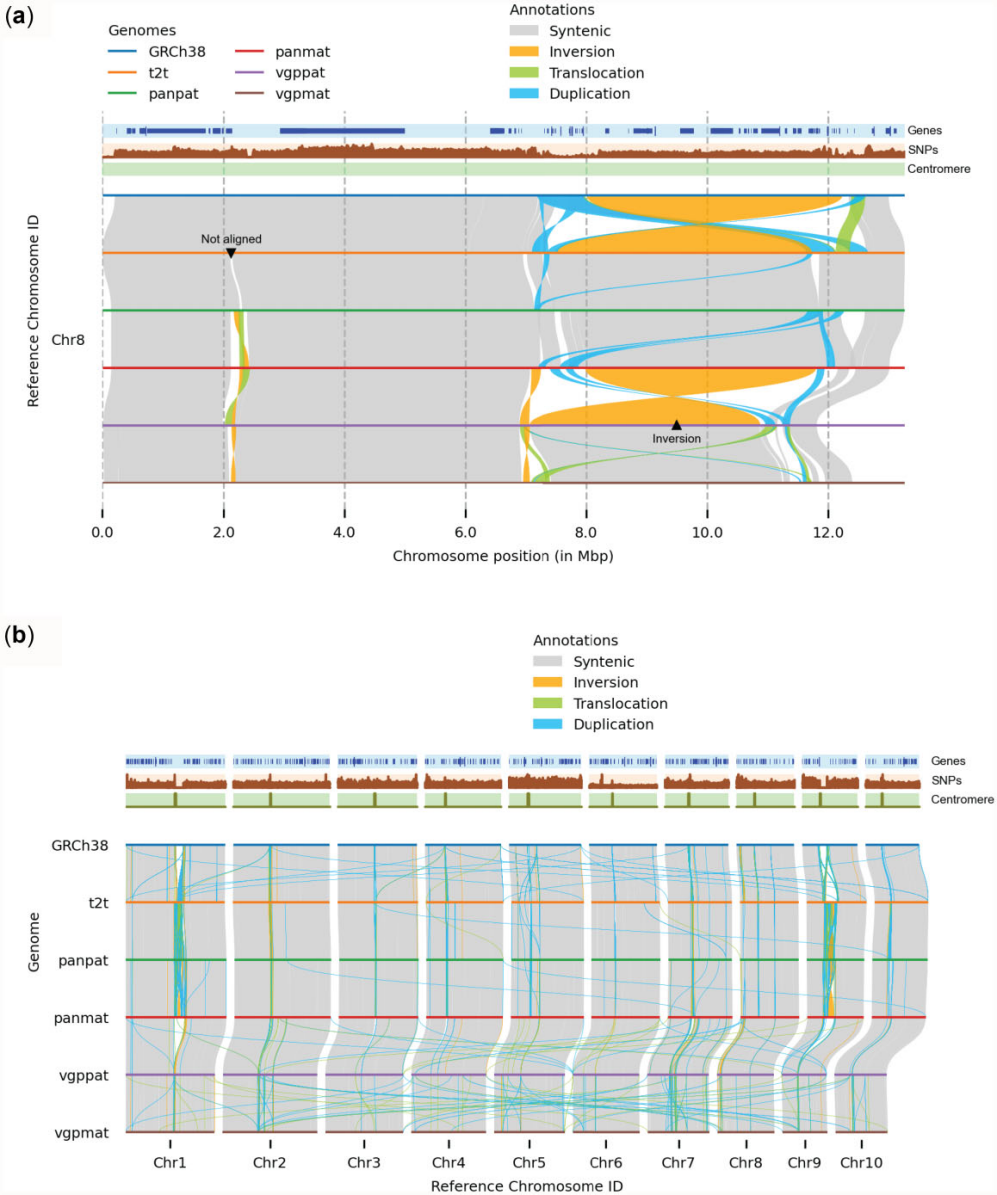
Customizing visualization using plotsr. The individual panels were created using plotsr without any further modifications. (**a**) Zooming in on a specific location allows for resolved visualization of the local genomic differences. Here, we visualized Chr8:1–13 000 000. Using plotsr, we have labelled a large inversion and a not-aligned region that became visible in the zoomed in view. (**b**) Inter-chromosomal rearrangements among the 10 chromosomes

plotsr is a python-based command-line tool. It requires the chromosome size (either through a fasta file or as a table) and the synteny and SRs information between the assemblies in a pairwise manner as input. For example, to visualize genomes A, B and C in this order, plotsr requires the comparison of A versus B and B versus C. These can be generated using genomic difference identification methods like SyRI, MUM&Co or assemblytics (Goel *et al.*, 2019; Nattestad and Schatz, 2016; O’Donnell and Fischer, 2020). The output of SyRI is accepted directly, while output from other methods can be provided in BEDPE format. Firstly, plotsr validates that the assemblies and structural information are consistent. Then, by using the pairwise synteny between genomes, it groups homologous chromosomes across the genomes and then plots the syntenic regions as well as SRs between them. plotsr can generate plots in two modes, (i) stacked mode: for better visualization of synteny and intra-chromosome rearrangements (Fig. 1); (ii) itx mode (similar to plots generated by JCVI): for better visualization of inter-chromosomal rearrangements (Fig. 2) (Tang *et al.*, 2015). The output can be generated in pdf, png or svg format. In addition, plotsr can show markers at predefined loci (e.g. genes, TEs or genomic markers) using BED files. plotsr can also plot the distribution of genomic features along the chromosomes (e.g. distribution of genes, SNPs, sequencing reads, etc.). This provides a visual comparison between sequence features and structural properties of the chromosomes. To adjust the plots, plotsr includes multiple parameters to control the visual properties (colour, size, spacing, etc.) of genomes, markers and tracks.

plotsr can also be used to zoom in on specific regions in any of the input genomes. For this, plotsr identifies the corresponding orthologous regions in all other genomes. This is a non-trivial task as some regions might include multiple rearrangements that obfuscate the syntenic regions in the other genomes. The identification of all syntenic regions would require whole-genome alignments of all genomes against the genome of interest implying the need for an all-versus-all genome alignment as input. This is computationally prohibitive once more than a few dozen genomes are involved. Instead, plotsr overcomes this challenge by using the syntenic backbone between the genomes to zoom in on any given region. For this, plotsr iteratively selects the regions syntenic to the selected region using pairwise genome comparisons until all genomes are covered. It then filters the structural information to only plot information overlapping these homologous regions resulting in a zoomed-in view of the genomes. Markers and feature tracks are also filtered automatically to plot those overlapping with the homologous regions.

## 3 Results

We visualized structural rearrangements between the human reference sequence (GRCh38), the human telomere-to-telomere assembly (t2t), two assemblies from the Human Pangenome Reference Consortium (panpat and panmat) and two assemblies from the Vertebrate Genomes Project (vgppat and vgpmat) using plotsr (Abdellah *et al.*, 2004; Jarvis *et al.*, 2022; Nurk *et al.*, 2021; Rhie *et al.*, 2021). Figure 1 shows the structural rearrangements in the first ten chromosomes whereas Supplementary Figure S1 shows structural rearrangements in all autosomal chromosomes. For this, pairwise whole-genome alignments were performed using minimap2 followed by synteny and structural rearrangement identification using SyRI (Goel *et al.*, 2019; Li, 2018). We also plotted gene annotation, distribution of common SNPs and centromere coordinates. Figure 1 shows that the genomes are predominantly syntenic (grey alignments). The vgppat and vgpmat assemblies have smaller pericentromeric regions (highly rearranged regions near the centromere) in chromosomes 1 and 9. Consequently, these chromosomes are smaller in vgppat and vgpmat genomes than other genomes. We also observed the depletion of genes in the centromeric regions. Using plotsr, we could also zoom in to highlight the genomic differences at Chr8:1–13 000 000 (reference genome coordinates) (Fig. 2a). The region was provided as a command-line parameter to plotsr which then automatically filtered and plotted the syntenic regions and rearrangements in all of the other genomes. In this region, we observed large inversions between the assemblies (labeled as ‘Inversion’ using plotsr) suggesting the presence of broadly two haplotypes (Logsdon *et al.*, 2021). We also observed that a large region without any alignment between the t2t and the panpat genomes (labelled as ‘Not aligned’) became visible within the zoom-in visualization. In Figure 2b, we show the inter-chromosomal translocations and duplications between the assemblies as well using the ‘itx mode’ visualization from plotsr.

We benchmarked plotsr by visualizing differences in six human (haploid genome size: ∼3 Gbp), eight *Arabidopsis thaliana* (haploid genome size: ∼120 Mbp, Supplementary Fig. S2) and four potato (haploid genome size: ∼800 Mbp, Supplementary Fig. S3) genomes. plotsr finished within 1 min and used less than 0.5 GB of RAM for all tests (Supplementary Fig. S4). Runtime and memory both scaled linearly with the number of samples. They were independent of the size of the genome, rather they were correlated to the number of structural rearrangements present in the genomes. Filtering out small variants (SNPs and InDels) from input files further improved the runtime in all tests. We also demonstrated the usability of plotsr with different structural differences identification methods by visualizing genomic differences between *A.thaliana* accessions identified by MUM&Co and assemblytics (Supplementary Figs S5 and S6, Supplementary Note S1) (Nattestad and Schatz, 2016; O’Donnell and Fischer, 2020).

## 4 Discussion and conclusion

The advent of long-read sequencing technologies has simplified the generation of high-quality genome assemblies. To support the visual analysis of such assemblies, we presented plotsr, a python-based command-line tool for visualizing structural similarities and rearrangements between genomes. In addition, plotsr allows visualization of genomic features as well as zoom-in views on specific regions. plotsr is highly efficient as it only requires pairwise comparisons in the order in which the genomes are compared. In turn, this limits the visualization flexibility because different orders of the genomes would require additional comparisons (which of course could be generated). However, often the genome order is predetermined (e.g. based on phylogeny), and in such cases, pairwise comparisons are computationally more efficient than comparing all genomes against each other.

plotsr generates publication-quality visualizations that have already been used by several research groups (Li *et al.*, 2021; van Rengs *et al.*, 2022; Zamyatin *et al.*, 2021; Zhang *et al.*, 2021). We believe that plotsr visualizations will help in getting a better understanding of the genome divergence of a species. Given the great importance of genomic analysis in many research fields, we are continuously developing plotsr to add more useful parameters allowing for more control and customization.

## Supplementary Material

btac196_Supplementary_Data
