# Correction to: plotsr: visualizing structural similarities and rearrangements between multiple genomes

**DOI:** 10.1093/bioinformatics/btac661

**Published:** 2022-10-13

This is a correction to: Manish Goel and Korbinian Schneeberger plotsr: visualizing structural similarities and rearrangements between multiple genomes, *Bioinformatics*, Volume 38, Issue 10, 15 May 2022, https://doi.org/10.1093/bioinformatics/btac196

In the originally published version of the manuscript, there were errors in the details of affiliations for both authors. The affiliations should read:

“^1^Department of Genetics, Faculty of Biology, LMU Munich, Großhaderner Str. 2, 82152 Planegg-Martinsried, Germany and ^2^Department of Chromosome Biology, Max Planck Institute for Plant Breeding Research, Carl-von-Linné-Weg 10, 50829 Cologne, Germany”

instead of:

“^1^Faculty of Biology, LMU Munich, Planegg-Martinsried 82152, Germany and ^2^Department of Genetics, Faculty of Biology, LMU Munich, Germany”.

The publisher apologizes for these errors. The errors have now been corrected within the article.

