# Supplementary material for: plotsr: visualizing structural similarities and rearrangements between multiple genomes
Source: Bioinformatics. 2022 Apr 15;38(10):2922–6. doi: 10.1093/bioinformatics/btac196 (PMC9113368; doi:10.1093/bioinformatics/btac196)
Supplement: btac196_Supplementary_Data [file btac196_supplementary_data.docx]

## Supplementary Figures


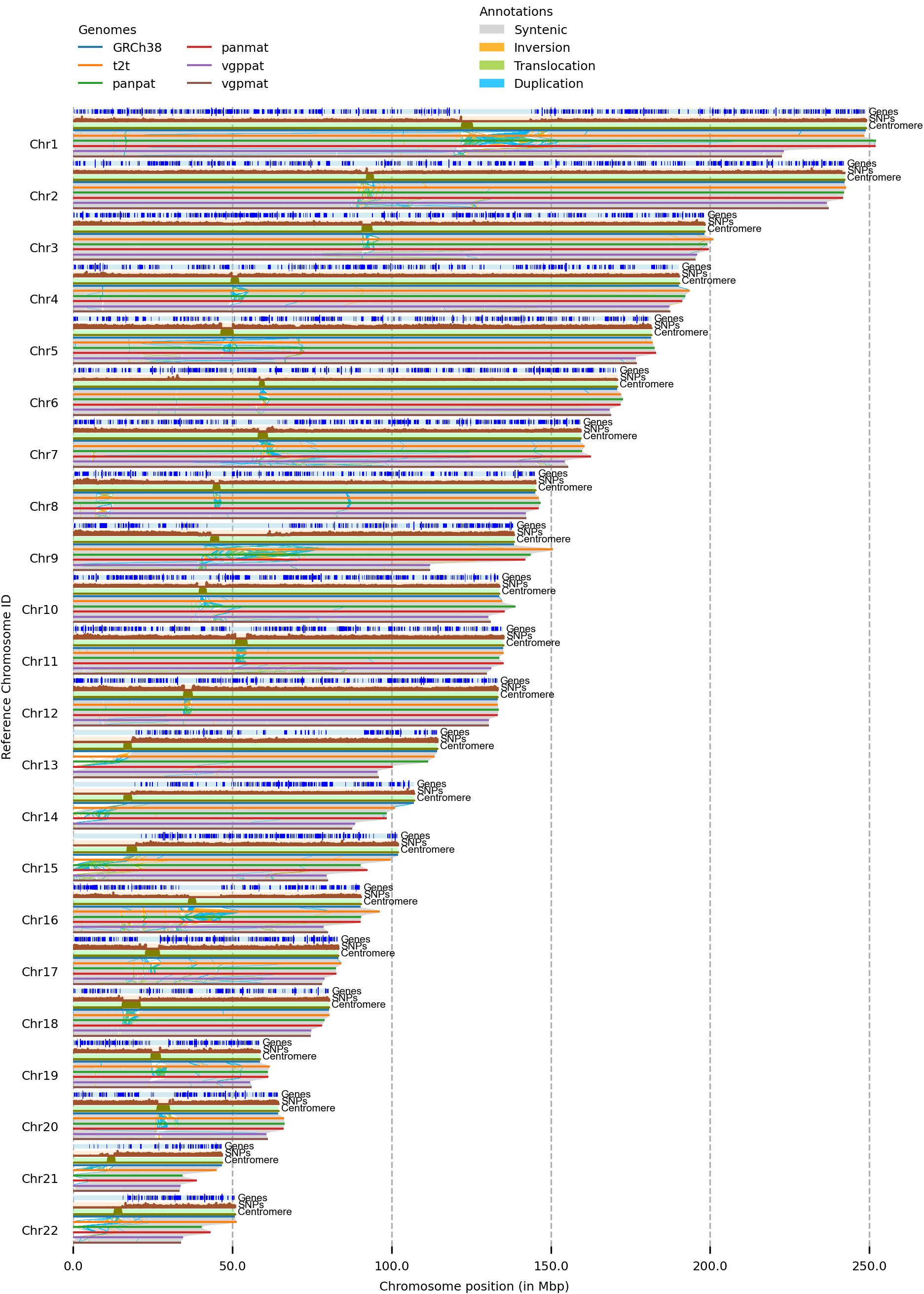


**Supplementary Figure 1:** plotsr visualization of syntenic regions and structural rearrangements between twelve chromosomes from six human assemblies. The visualisation was created using plotsr without further modifications. Tracks for three genomic features: genes, number of SNPs, and centromeric regions were included using optional parameters. In the genes track, small blue lines correspond to transcribed regions and long blues lines represent coding sequences (CDS).


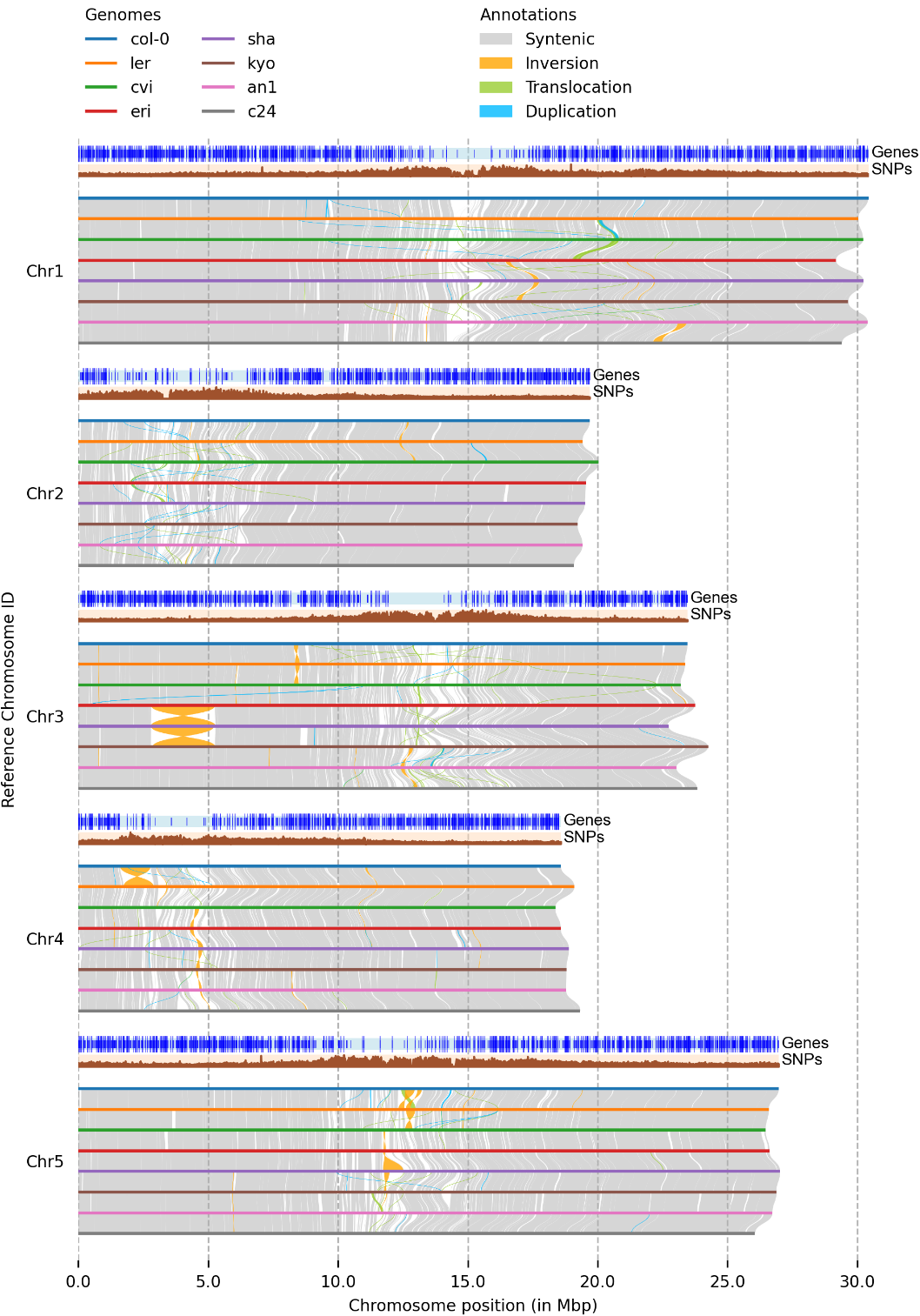

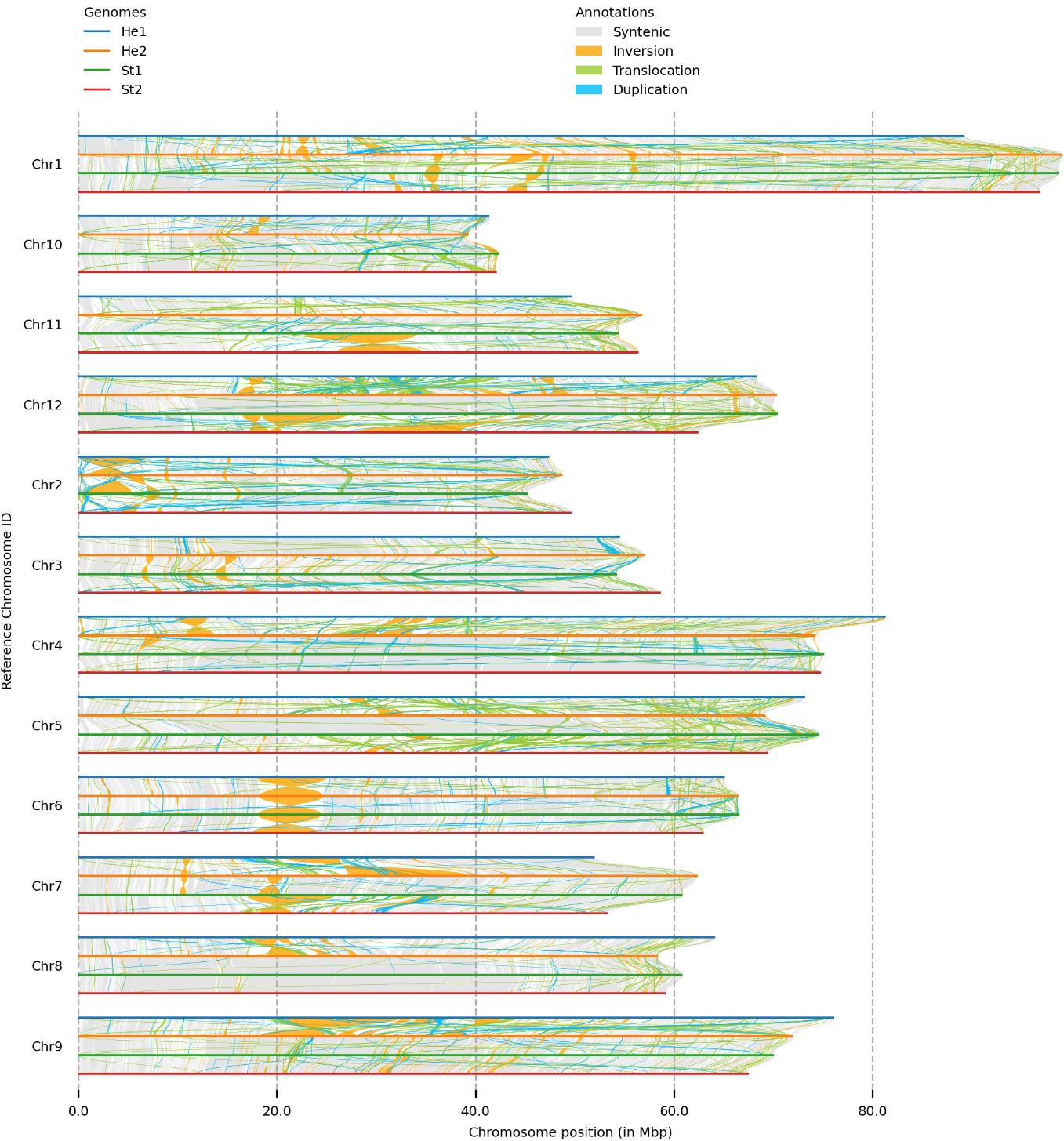


**Supplementary Figure 2:** plotsr visualization of syntenic regions and structural rearrangements between eight *Arabidopsis thaliana* genome assemblies (Jiao & Schneeberger, 2020; The Arabidopsis Genome Initiative, 2000) . The visualisation was created using plotsr without further modifications. Tracks for three genomic features: genes, number of SNPs, and centromeric regions were included using optional parameters (Alonso-Blanco et al., 2016; Giraut et al., 2011; Lamesch et al., 2012). In the genes track, small blue lines correspond to transcribed regions and long blues lines represent coding sequences (CDS).

**Supplementary Figure 3:** plotsr visualisation of syntenic regions and structural rearrangements between four haplotypes of the potato cultivar ‘Otava’ (Sun et al., 2022). The visualisation was created using plotsr without further modifications.


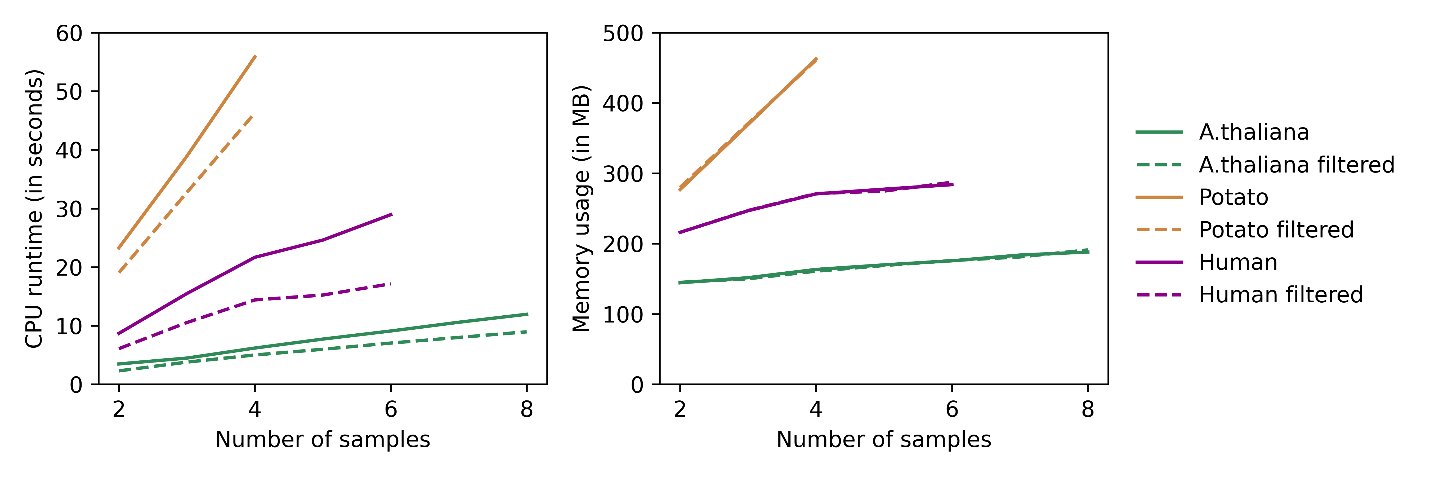


**Supplementary Figure 4:** Benchmarks for CPU and memory usage by plotsr. Tests were done on a desktop using Debian-9.13 with Intel i5-6600 CPU (3.3 GHz) and 16GB of RAM. Solid lines use the raw output from SyRI, the dashed line uses output files from which SNPs and Indels have been filtered out.


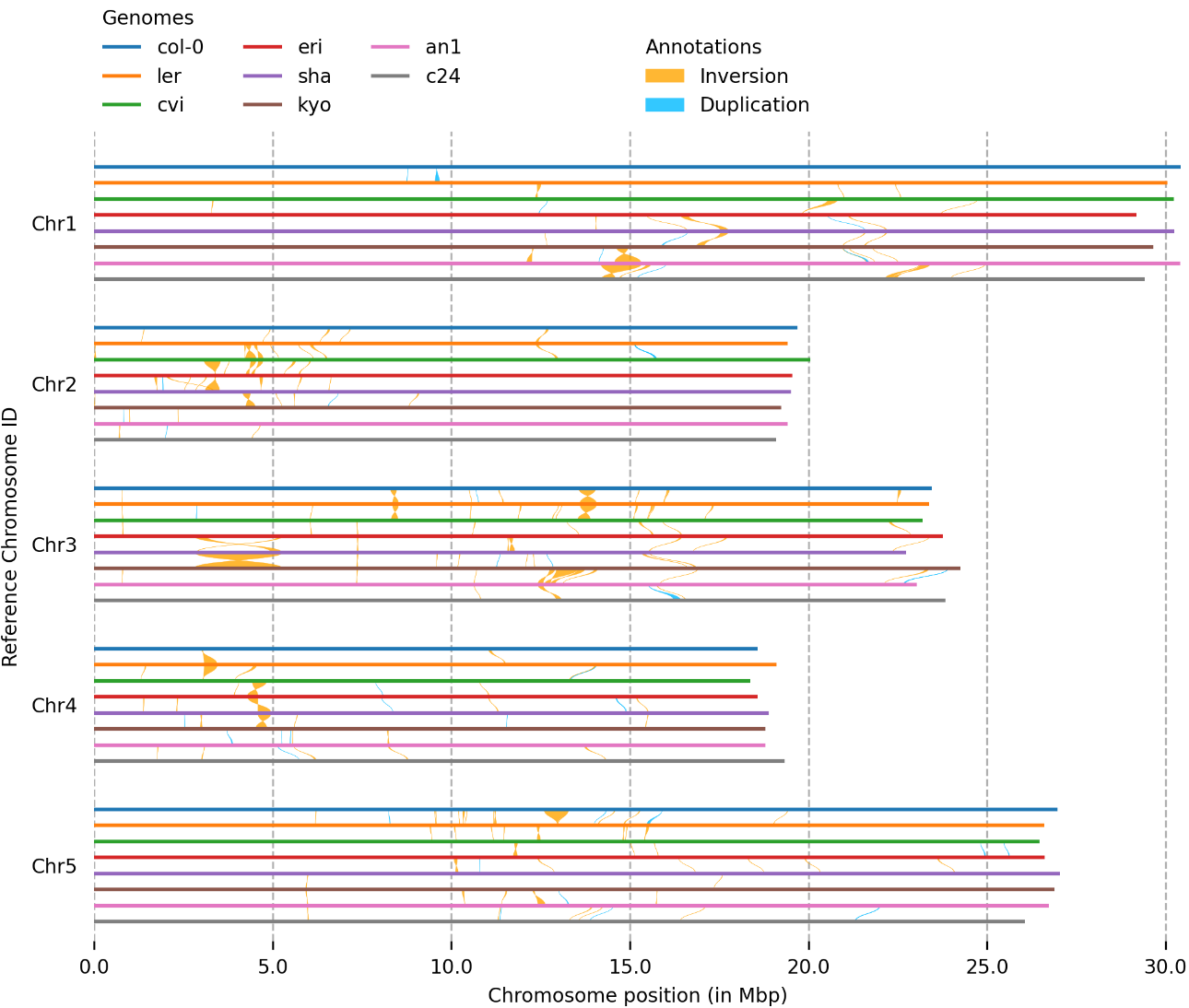


**Supplementary Figure 5:** plotsr visualisation of structural rearrangements identified by MUM&Co between eight *Arabidopsis thaliana* genome assemblies. The visualisation was created using plotsr without further modifications.


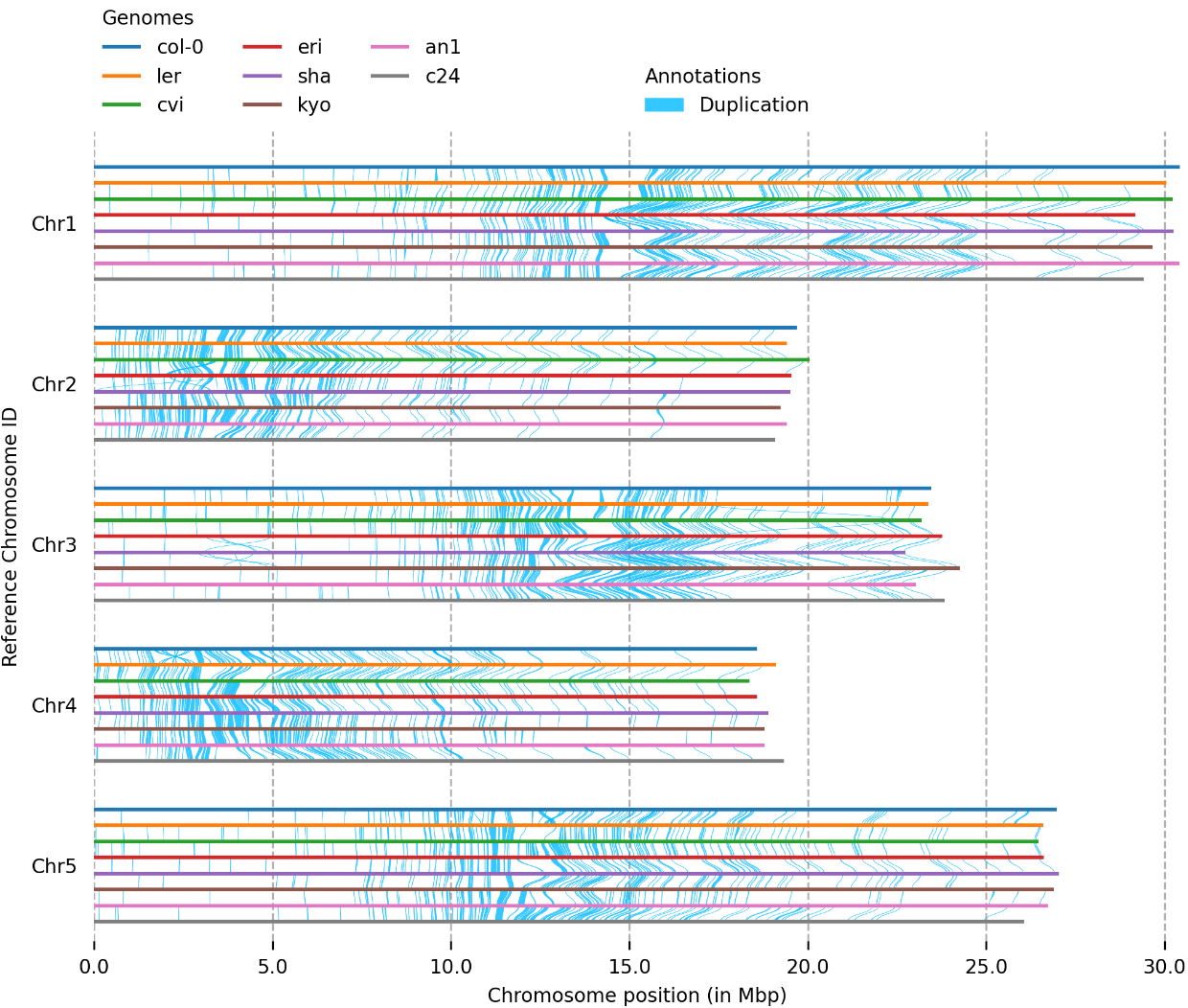


**Supplementary Figure 6:** plotsr visualisation of structural rearrangements identified by Assemblytics between eight *Arabidopsis thaliana* genome assemblies. The visualisation was created using plotsr without further modifications.

## Supplementary Note 1

For visualising structural rearrangements identified by MUM&co and Assemblytics, we first identified pairwise SRs between eight *A. thaliana* strains and then transformed the output from these tools to get SRs in a BEDPE format. Below are the code-snippets for converting MUM&co and Assemblytics output from the *A. thaliana* comparison to BEDPE format. Similar steps can be done for other samples and SR identification methods.

- Pro-processing MUM&co output.

# Iterate over all pairwise comparisons

for s in col_ler ler_cvi cvi_eri eri_sha sha_kyo kyo_an1 an1_c24 ; do

# Remove header

tail +2 ${s}_output/${s}.SVs_all.tsv \

# Select variants

| grep -E 'duplication|inversion' \

# Select columns containing ref_chr, ref_start, ref_end, qry_chr, qry_start, qry_end, sr_type and save to BEDPE file

| awk '{print $1"\t"$3"\t"$4"\t"$2"\t"$7"\t"$8"\t"$6}' > ${s}.svs.bed

# Change SR name to compatible strings

sed -i 's/duplication/DUP/g;s/inversion/INV/g' ${s}.svs.bed

# Add fake syntenic regions, these will not be plotted

echo -e 'Chr1\t1\t29000000\tChr1\t1\t29000000\tSYN' >> ${s}.svs.bed

echo -e 'Chr2\t1\t19000000\tChr2\t1\t19000000\tSYN' >> ${s}.svs.bed

echo -e 'Chr3\t1\t22000000\tChr3\t1\t22000000\tSYN' >> ${s}.svs.bed

echo -e 'Chr4\t1\t18000000\tChr4\t1\t18000000\tSYN' >> ${s}.svs.bed

echo -e 'Chr5\t1\t26000000\tChr5\t1\t26000000\tSYN' >> ${s}.svs.bed

# Sort the BEDPE file

sort -k1,1 -k2,2n ${s}.svs.bed > ${s}.svs.sorted.bed

done

# Visualise the structural rearrangments

plotsr --nosyn --bp col_ler.svs.sorted.bed --bp ler_cvi.svs.sorted.bed --bp cvi_eri.svs.sorted.bed --bp eri_sha.svs.sorted.bed --bp sha_kyo.svs.sorted.bed --bp kyo_an1.svs.sorted.bed --bp an1_c24.svs.sorted.bed --genomes genomes.txt -o plotsr_with_mumnco.png -W 8 -H 6 -f 8 --cfg base.cfg

- Pro-processing Assemblytics output.

# Iterate over all pairwise comparisons

for s in col_ler ler_cvi cvi_eri eri_sha sha_kyo kyo_an1 an1_c24 ; do

# Remove header

tail +2 ${s}.Assemblytics_structural_variants.bed \

# Select variants

| grep -E 'Repeat_contraction|Repeat_expansion|Tandem_contraction|Tandem_expansion' \

# Select columns containing ref_chr, ref_start, ref_end, qry_chr, qry_start, qry_end, sr_type and save to BEDPE file

| awk '{split($10,a,":"); print $1"\t"$2"\t"$3"\t"a[1]"\t"a[2]"\tDUP"}' > ${s}.svs.bed

sed -i 's/-/\t/g' ${s}.svs.bed

# Add fake syntenic regions, these will not be plotted

echo -e 'Chr1\t1\t29000000\tChr1\t1\t29000000\tSYN' >> ${s}.svs.bed

echo -e 'Chr2\t1\t19000000\tChr2\t1\t19000000\tSYN' >> ${s}.svs.bed

echo -e 'Chr3\t1\t22000000\tChr3\t1\t22000000\tSYN' >> ${s}.svs.bed

echo -e 'Chr4\t1\t18000000\tChr4\t1\t18000000\tSYN' >> ${s}.svs.bed

echo -e 'Chr5\t1\t26000000\tChr5\t1\t26000000\tSYN' >> ${s}.svs.bed

# Sort the BEDPE file

sort -k1,1 -k2,2n ${s}.svs.bed > ${s}.svs.sorted.bed

done

# Visualise the structural rearrangments

plotsr --nosyn --bp col_ler.svs.sorted.bed --bp ler_cvi.svs.sorted.bed --bp cvi_eri.svs.sorted.bed --bp eri_sha.svs.sorted.bed --bp sha_kyo.svs.sorted.bed --bp kyo_an1.svs.sorted.bed --bp an1_c24.svs.sorted.bed --genomes genomes.txt -o plotsr_with_assemblytics.png -W 8 -H 6 -f 8 --cfg base.cfg

## Bibliography

Alonso-Blanco, C., Andrade, J., Becker, C., Bemm, F., Bergelson, J., Borgwardt, K. M. M., … Zhou, X. (2016). 1,135 Genomes Reveal the Global Pattern of Polymorphism in Arabidopsis thaliana. *Cell*, *166*(2), 481–491. https://doi.org/10.1016/j.cell.2016.05.063

Giraut, L., Falque, M., Drouaud, J., Pereira, L., Martin, O. C., & Mézard, C. (2011). Genome-wide crossover distribution in Arabidopsis thaliana meiosis reveals sex-specific patterns along chromosomes. *PLoS Genetics*, *7*(11), e1002354. https://doi.org/10.1371/journal.pgen.1002354

Jiao, W.-B., & Schneeberger, K. (2020). Chromosome-level assemblies of multiple Arabidopsis genomes reveal hotspots of rearrangements with altered evolutionary dynamics. *Nature Communications*, *11*(1), 989. https://doi.org/10.1038/s41467-020-14779-y

Lamesch, P., Berardini, T. Z., Li, D., Swarbreck, D., Wilks, C., Sasidharan, R., … Huala, E. (2012). The Arabidopsis Information Resource (TAIR): improved gene annotation and new tools. *Nucleic Acids Research*, *40*(D1), D1202–D1210. https://doi.org/10.1093/nar/gkr1090

Sun, H., Jiao, W.-B., Krause, K., Campoy, J. A., Goel, M., Folz-Donahue, K., … Schneeberger, K. (2022). Chromosome-scale and haplotype-resolved genome assembly of a tetraploid potato cultivar. *Nature Genetics 2022*, 1–7. https://doi.org/10.1038/s41588-022-01015-0

The Arabidopsis Genome Initiative. (2000). Analysis of the genome sequence of the flowering plant Arabidopsis thaliana. *Nature*, *408*(6814), 796–815. https://doi.org/10.1038/35048692

Willing, E. M., Piofczyk, T., Albert, A., Winkler, J. B., Schneeberger, K., & Pecinka, A. (2016). UVR2 ensures transgenerational genome stability under simulated natural UV-B in Arabidopsis thaliana. *Nature Communications*, *7*, 1–9. https://doi.org/10.1038/ncomms13522
